# Influence of lesion and disease subsets on the diagnostic performance of the quantitative flow ratio in real-world patients

**DOI:** 10.1038/s41598-021-82235-y

**Published:** 2021-02-04

**Authors:** Kwan Yong Lee, Byung-Hee Hwang, Moo Jun Kim, Eun-Ho Choo, Ik Jun Choi, Chan Jun Kim, Sang-Wook Lee, Joo Myung Lee, Mi-Jeong Kim, Doo Soo Jeon, Wook Sung Chung, Ho-Joong Youn, Ki Jun Kim, Myeong-Ho Yoon, Kiyuk Chang

**Affiliations:** 1 Department of Cardiology, Incheon St. Mary’s Hospital, The Catholic University of Korea, Incheon, Republic of Korea; 2 Department of Cardiology, Seoul St. Mary’s Hospital, The Catholic University of Korea, 222, Banpo-daero, Seocho-gu, Seoul, 06591 Republic of Korea; 3 Cardiovascular Research Institute, Seoul St. Mary’s Hospital, The Catholic University of Korea, Seoul, Republic of Korea; 4 Department of Cardiology, Uijeongbu St. Mary’s Hospital, The Catholic University of Korea, Uijeongbu, Republic of Korea; 5 School of Mechanical Engineering, University of Ulsan, Ulsan, Republic of Korea; 6 Department of Cardiology, Department of Internal Medicine, Heart Vascular Stroke Institute, Samsung Medical Center, Sungkyunkwan University School of Medicine, Seoul, Republic of Korea; 7 Department of Radiology, Incheon St Mary’s Hospital, The Catholic University of Korea, Incheon, Republic of Korea; 8 Department of Cardiology, Ajou University Medical Center, Suwon, Republic of Korea

**Keywords:** Interventional cardiology, Atherosclerosis, Computational biophysics

## Abstract

The quantitative flow ratio (QFR) is a novel angiography-based computational method assessing functional ischemia caused by coronary stenosis. This study aimed to evaluate the diagnostic performance of quantitative flow ratio (QFR) in patients with angina and acute myocardial infarction (AMI) and to identify the conditions with low diagnostic performance. We assessed the QFR for 1077 vessels under fractional flow ratio (FFR) evaluation in 915 patients with angina and AMI. The diagnostic accuracies of the QFR for identifying an FFR ≤ 0.8 were 95.98% (95% confidence interval [CI] 94.52 to 97.14%) for the angina group and 92.42% (95% CI 86.51 to 96.31%) for the AMI group. The diagnostic accuracy of the QFR in the borderline FFR zones (> 0.75, ≤ 0.85) (91.23% [95% CI 88.25 to 93.66%]) was significantly lower than that in others (difference: 4.32; p = 0.001). The condition accompanying both AMI and the borderline FFR zone showed the lowest QFR diagnostic accuracy in our data (83.93% [95% CI 71.67 to 92.38]). The diagnostic accuracy was reduced for tandem lesions (p = 0.04, not correcting for multiple testing). Our study found that the QFR method yielded a high overall diagnostic performance in real-world patients. However, low diagnostic accuracy has been observed in borderline FFR zones with AMI, and the hybrid FFR approach needs to be considered.

## Introduction

Strong evidence indicates that the fractional flow ratio (FFR) can identify functional myocardial ischemia in intermediate-degree coronary stenotic lesions^1–4^. In addition, revascularization guided by the FFR is superior to revascularization guided by angiography in terms of improving clinical outcomes in patients with stable angina (SA)^5–8^. Therefore, the FFR is being increasingly utilized in clinical practice; however, the possible risks during FFR measurement, including the need for hyperemia-inducing drugs and an invasive pressure-wire insertion procedure, may contribute to the low application of the FFR in real-world catheterization laboratories^9,10^. Accordingly, the quantitative flow ratio (QFR) is a fascinating assessment tool for measuring functional ischemia in coronary stenosis or vessels based on coronary angiography (CAG) without the need for induced hyperemia and pressure wire usage. The QFR is computed from CAG based on 3D reconstruction and fluid dynamics algorithms using a modified frame count analysis^11^.

FFR is an indicator of the pressure difference between the stenotic lesion at a state with minimal microvascular resistance, measured in a drug-induced hyperemia state. Theoretically, for QFR to mimic FFR, frame count analysis on hyperemic angiogram should be applied (adenosine QFR), but QFR uses its own algorithm to infer this only by measuring the velocity of contrast agent at resting state without inducing hyperemia (contrast-flow QFR)^12^. An initial small study, the FAVOR II pilot study, showed that contrast-flow QFR exhibits similar accuracy to the adenosine QFR and is superior to fixed-flow QFR based on a fixed empiric hyperemic flow velocity of 0.35 m/s^12^. Since then, several studies have been performed and have successfully validated the diagnostic accuracy of contrast-flow QFR against adenosine FFR^13–16^. In the FAVOR II study from China, QFR was assessed online in real-time in a catheterization laboratory and yielded a diagnostic accuracy of 92.4% relative to that of FFR^15^. However, the data were derived from limited small studies that excluded patients with severe comorbidities and vessels with complex lesions.

This study aimed to evaluate the diagnostic performance of the QFR against the FFR in a real-world all-comer population with angina and acute myocardial infarction (AMI) including vessels with complex high-risk lesions and to identify the conditions with low diagnostic accuracy.

## Methods

### Study population

**Figure 1:**
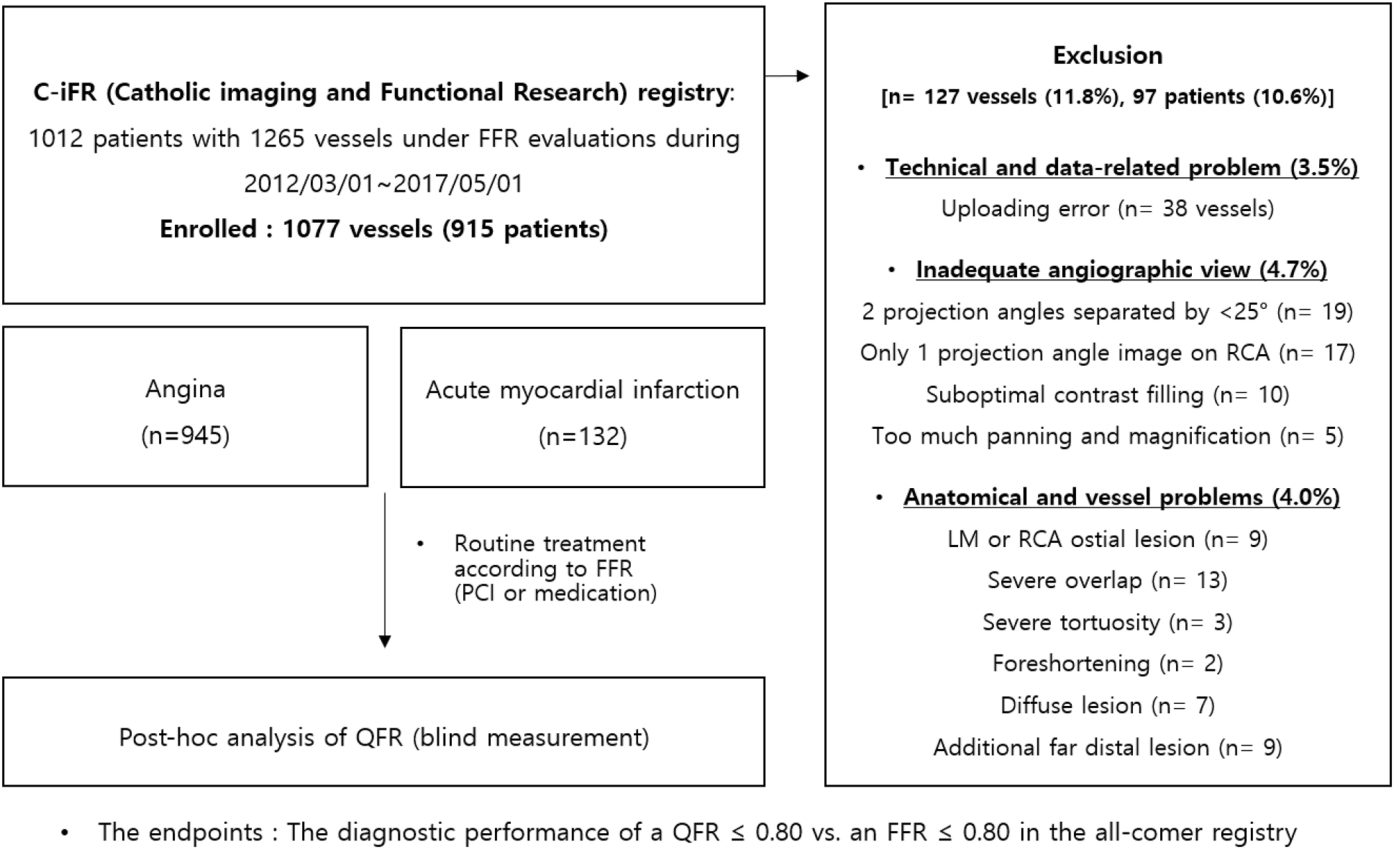
Flow chart of the study. *FFR* indicates fractional flow reserve, *LM* left main artery, *PCI* percutaneous coronary intervention, *QFR* quantitative flow ratio, *RCA* right coronary artery.

The Catholic Imaging and Functional Research (C-iFR) Cohort (NCT04102917) was designed to evaluate the diagnostic performance and clinical outcome predictive ability of QFR in consecutive patients undergoing CAG and FFR at 4 major cardiac centers affiliated with the Catholic University of Korea (Seoul St. Mary’s Hospital, Seoul; St. Paul’s Hospital, Seoul; Incheon St. Mary’s Hospital, Incheon; Uijeongbu St. Mary’s Hospital, Uijeongbu) from January 2012 to May 2018. A total of 1012 patients, including 1265 vessels, were registered in this observational registry. This registry includes demographic characteristics, clinical information, laboratory data, QFR findings, and FFR findings, with clinical outcome data collected over a median of 2.29 [1.15, 3.36] years. FFR tests were conducted on all-comer patients with angina and AMI, which clinicians determined to be intermediate stenosis that visually indicated physiological lesions in CAG tests. The actual mean percent area stenosis measured at post-hoc analysis was 64.65 ± 9.72%, and 65.2% of vessels showed 50 ~ 70% stenosis. The distribution of more detailed stenosis degrees is expressed in the histogram of supplementary Fig. 1. In patients with AMI, the FFR was measured only in noninfarct-related arteries. A flow chart of the study population is depicted in Fig. 1. We analyzed QFR for a total of 1077 vessels from 915 patients after exclusion of 97 patients and 127 vessels with calibration failure for the following reasons: data uploading errors (3.5% of all vessels); insufficient angiographic views for analysis (4.7%), including cases containing 2 projection angles < 25 degrees apart, only 1 projection angle image for the right coronary artery, images with suboptimal contrast filling, and images with too much panning or too much magnification; and anatomical vessel problems (4.0%), including cases containing an ostial lesion of the left main coronary artery or right coronary artery, severe overlap, severe tortuosity, foreshortening, diffuse lesions, and an additional far distal lesion. No corporations were involved in the design, performance, or data analysis of the study. This observational study was approved by the Catholic Medical Center Central Institutional Review Board (IRB) and each participating hospital IRB. It was performed in accordance with the Strengthening the Reporting of Observational Studies in Epidemiology guidelines^17^. We received an informed consent waiver from the Catholic Medical Center Central IRB and each participating hospital IRB because this retrospective study using medical records involves no more than minimal risk to subjects.

### CAG, quantitative coronary analysis (QCA) and QFR assessment

CAG projections were recorded at 15 frames/s by a monoplane radiographic system (Siemens, Philips, Toshiba) after the administration of intracoronary nitroglycerin (100 or 200 mcg). The contrast medium was injected using an automatic injecting device at a rate of 3 to 4 ml/s. 3D-QCA was performed, and the QFR was measured with QAngio XA 3D/QFR, V 1.2 by Medis Medical Imaging, Leiden, The Netherlands. All angiographic images and FFR data were sent to the core laboratory at Incheon Saint Mary’s Hospital. QCA and QFR analyses were performed in a blinded fashion without information on the FFR value. QFR analysis was performed by a well-trained technician and physician who had previously performed QFR analyses in approximately 300 cases.

### QFR analysis

The QFR was calculated on the basis of 3D reconstruction and fluid dynamics algorithms using a modified frame count analysis. After selecting two angiographic projection images with different views separated by angles of at least 25 degrees, indication and offset corrections of the proximal and distal reference points were performed, including the stenotic lesion of interest. Then, the software performed 3D reconstruction after detecting and adding path lines and contours to the target vessel and lesion. The 3D reconstructed vessel segment was automatically divided into several subsegments, and the pressure drop at every position was calculated by integrating the pressure drop of all subsegments. A previously reported function was applied to quantify the delta pressure using the calculated hyperemic flow velocity relationship assumed by the measured contrast flow velocity in nonhyperemic conditions^12^. The pressure drop function considered viscosity and flow separation. Finally, we assessed the contrast-flow QFR, which is a pressure drop value based on contrast flow velocity using frame count analysis. The detailed physiologic and computational algorithms of the QFR have been described previously^11,12^. Quantified values such as lesion length, flow rate, and plaque volume were also measured by QFR software.

### Invasive procedures

The FFR measurements were obtained using a pressure wire (Philips Volcano, San Diego, California, USA or Abbott St. Jude Medical. St. Paul. Minnesota, USA). Only lesions with 50 to 70% narrowing on visual examination by CAG were eligible to be measured with the physiologically guided FFR assessment. After calibration, equalization and placement of the pressure wire, we administered an intracoronary injection of nitroglycerin before the FFR assessment. Intravenous adenosine triphosphate (140 mcg/kg/min over at least 1.5 min) infusion or an intracoronary injection of nicorandil (2 mg for left coronary artery, 1.5 mg for right coronary artery) was administered to induce hyperemia to measure the FFR. After the FFR assessment, the pressure wire was returned to the tip of the guide catheter to avoid pressure drift. Patients without a limited drift and with values between 0.98 and 1.02 were included.

### Study endpoints and follow-up

The primary endpoints were the accuracy, sensitivity, specificity, positive predictive value (PPV), and negative predictive value (NPV) of QFR ≤ 0.8 for identifying FFR ≤ 0.8 as the reference standard. We compared the diagnostic performance in subgroups with angina, AMI, a borderline FFR (≥ 0.75, ≤ 0.85) and complicated coronary lesions, such as bifurcation lesions, a large intraluminal plaque volume, a low flow velocity, a long lesion length, calcification, tandem lesions, and a previous history of coronary intervention.

### Statistical analysis

The baseline demographics, vessel characteristics and biochemical characteristics are summarized as the mean ± standard deviation for continuous variables and as absolute numbers and percentages for discrete variables. Data were analyzed on a per-patient basis for clinical characteristics and on a per-vessel basis for the remaining calculations. Differences in continuous variables between groups were evaluated using the Wilcoxon rank-sum test. Differences in discrete variables between groups were analyzed using χ^2^ or Fisher’s exact tests. The distributions of the FFR, QFR, and PAS (percent area stenosis) by 3D QCA values are described in the frequency histograms. Correlations and agreement between the FFR and QFR values were assessed by Pearson’s correlation coefficients and Bland–Altman plots. The diagnostic sensitivity, specificity, PPV, NPV, accuracy, and area under the receiver operating characteristic curve (AUC) were calculated using an FFR ≤ 0.8. The diagnostic accuracies (percent agreements) were compared across groups by the chi-square test or Fisher's exact test. Each measure was analyzed using SAS 9.4 (SAS Institute, Cary, NC, USA). Statistical significance was indicated by a two-tailed p < 0.05.

## Results

### Characteristics of the patients and target vessels

The baseline clinical characteristics, vessel characteristics and physiologic data between the angina and AMI groups are described in Table 1. The calculated mean Framingham risk score of all patients was 12.1 ± 8.4, categorized in the intermediate future cardiovascular risk group. Among them, 40.2% and 71.7% had type 2 diabetes and hypertension, respectively. In patients with AMI, more smokers, lower left ventricle systolic function, lower eGFR, and higher total cholesterol and low-density cholesterol levels were observed (p < 0.05, each). Multivessel disease and calcified and tandem lesions were more common in the AMI group (p < 0.05, each). The percent area stenosis was significantly higher in the AMI group than in the angina group (p < 0.001, both). However, there were no significant differences in intraluminal plaque volume, flow velocity or lesion length between the groups (p = 0.235, p = 0.532, and p = 0.736, respectively). Ultimately, after FFR evaluation, revascularization of target vessels was performed for 42.8% of the vessels in the angina group and 66.7% of the noninfarct-related arteries in the AMI group.

**Table 1:** Baseline clinical characteristics, baseline results of the lesion analysis and physiologic outcomes among the groups distributed by clinical presentation.

	Total	Angina	AMI	p-value
Per-patient analysis	n = 915	n = 812	n = 103	
Age, year	67.0 (59.0–74.0)	67.0 (60.0–74.0)	66.0 (57.0–75.0)	0.121
Male	601 (65.7)	531 (65.4)	70 (68.0)	0.605
BMI	24.7 (22.7–26.7)	24.8 (22.8–26.7)	23.9 (22.1–25.6)	0.010
DM	368 (40.2)	320 (39.4)	48 (46.6)	0.161
HBP	655 (71.7)	584 (72.0)	71 (68.9)	0.514
Dyslipidemia	514 (56.2)	473 (58.3)	41 (39.8)	< 0.001
CKD	92 (10.1)	72 (8.9)	20 (19.4)	0.001
CVA	71 (7.8)	61 (7.5)	10 (9.7)	0.433
Smoker	350 (38.3)	291 (35.9)	59 (57.3)	< 0.001
Family history of CAD	49 (5.4)	44 (5.4)	5 (4.9)	0.809
Previous MI	79 (8.6)	62 (7.6)	17 (16.5)	0.003
Previous PCI	259 (28.3)	232 (28.6)	27 (26.2)	0.617
Previous CABG	7 (0.8)	7 (0.9)	0 (0.0)	> 0.999
Framingham score	11.0 (5.5–17.0)	11.2 (5.5–17.0)	10.5 (5.5–17.8)	0.878
SBP	124.0 (110.0–140.0)	124.0 (110.0–140.0)	130.0 (114.0–146.0)	0.352
DBP	75.0 (67.0–80.0)	75.0 (67.0–80.0)	74.0 (67.0–80.0)	0.775
LVEF	61.0 (56.0–64.8)	61.2 (57.0–65.0)	56.0 (45.0–63.0)	< 0.001
Hemoglobin	13.2 (11.8–14.5)	13.2 (11.8–14.5)	13.2 (11.3–14.9)	0.721
eGFR	75.2 (60.2–89.9)	75.5 (61.9–89.9)	73.1 (49.5–90.0)	0.121
hsCRP	0.4 (0.1–1.5)	0.3 (0.1–1.1)	1.6 (0.3–18.2)	< 0.001
Total cholesterol	151.0 (130.0–181.0)	150.0 (130.0–178.0)	166.0 (130.0–190.0)	0.066
Triglyceride	118.0 (81.0–166.0)	118.0 (81.0–168.0)	109.0 (78.0–154.0)	0.362
HDL	41.0 (35.0–48.0)	41.0 (35.0–48.0)	41.5 (35.0–49.5)	0.387
LDL	86.0 (68.0–109.0)	85.5 (68.0–107.0)	94.0 (70.0–123.0)	0.035
HbA1C	6.2 (5.7–7.1)	6.2 (5.7–7.1)	6.3 (5.6–7.4)	0.794
Dyslipidemia medication	643 (70.4)	589 (72.6)	54 (52.4)	< 0.001
DM medication	320 (35.1)	278 (34.4)	42 (40.8)	0.199
HBP medication	675 (73.9)	600 (74.0)	75 (72.8)	0.800
Aspirin medication	560 (61.3)	513 (63.3)	47 (45.6)	0.001
Other anti-platelet agents	443 (48.5)	402 (49.6)	41 (39.8)	0.060
Revascularization	294 (44.7)	258 (42.8)	36 (66.7)	0.001
Per-vessel analysis	n = 1077	n = 945	n = 132	
**Vessel characteristics**				
LAD (%)	705 (65.5)	632 (66.9)	73 (55.3)	0.016
LCX (%)	166 (15.4)	137 (14.5)	29 (22.0)	
LM (%)	19 (1.8)	14 (1.5)	5 (3.8)	
RCA (%)	187 (17.4)	162 (17.1)	25 (18.9)	
Multivessel disease	451 (63.0)	404 (61.1)	47 (85.5)	< 0.001
Vessel with a prior stent	146 (20.4)	134 (20.3)	12 (21.8)	0.785
Bifurcation lesion	748 (69.5)	660 (69.9)	88 (66.7)	0.448
Tortuosity	19 (1.8)	15 (1.6)	4 (3.0)	0.277
Calcified lesion	386 (35.9)	328 (34.7)	58 (43.9)	0.039
Thrombotic lesion	1 (0.1)	0 (0.0)	1 (0.8)	0.123
Tandem lesion	431 (40.1)	368 (39.0)	63 (48.1)	0.046
**Physiological parameters and quantitative coronary angiography**				
FFR	0.83 (0.77–0.89)	0.84 (0.77–0.89)	0.80 (0.75–0.87)	0.001
QFR	0.83 (0.77–0.89)	0.84 (0.77–0.89)	0.80 (0.75–0.86)	< 0.001
Percent area of stenosis, %	65.10 (59.10–70.70)	64.70 (59.10–70.60)	66.45 (59.85–71.90)	0.118
Reference diameter, mm	2.60 (2.30–2.90)	2.60 (2.30–2.90)	2.60 (2.20–3.00)	0.687
Minimal lumen diameter, mm	1.30 (1.10–1.60)	1.30 (1.10–1.60)	1.40 (1.10–1.60)	0.681
Intraluminal plaque volume, mm^3^	36.40 (22.60–58.30)	35.90 (22.30–57.50)	37.85 (23.80–65.00)	0.235
Flow velocity, m/s	0.21 (0.15–0.28)	0.21 (0.15–0.28)	0.21 (0.15–0.28)	0.532
Lesion length, mm	20.15 (13.95–30.05)	20.15 (14.00–29.85)	20.35 (13.85–31.85)	0.736

Data are presented as the n (%) for categorical variables and median (IQR) for continuous variables. The p-values for differences were determined using the chi-square test, Fisher's exact test or the Wilcoxon rank-sum test.

*AMI* acute myocardial infarction, *BMI* body mass index, *DM* diabetes mellitus, *HBP* high blood pressure, *CKD* chronic kidney disease, *CVA* cerebrovascular accident, *CAD* coronary artery disease, *MI* myocardial infarction, *PCI* percutaneous coronary intervention, *CABG* coronary artery bypass graft, *SBP* systolic blood pressure, *DBP* diastolic blood pressure, *LVEF* left ventricle ejection fraction, *eGFR* estimated glomerular filtration rate, *hsCRP* high sensitivity C-reactive protein, *HDL* high density lipoprotein, *LDL* low density lipoprotein, *LAD* left anterior descending artery, *LCX* left circumflex artery, *LM* left main artery, *RCA* right coronary artery, *FFR* fractional flow reserve, *QFR* quantitative flow ratio.

### Correlations, agreement and diagnostic performance of the QFR in the total population

**Figure 2:**
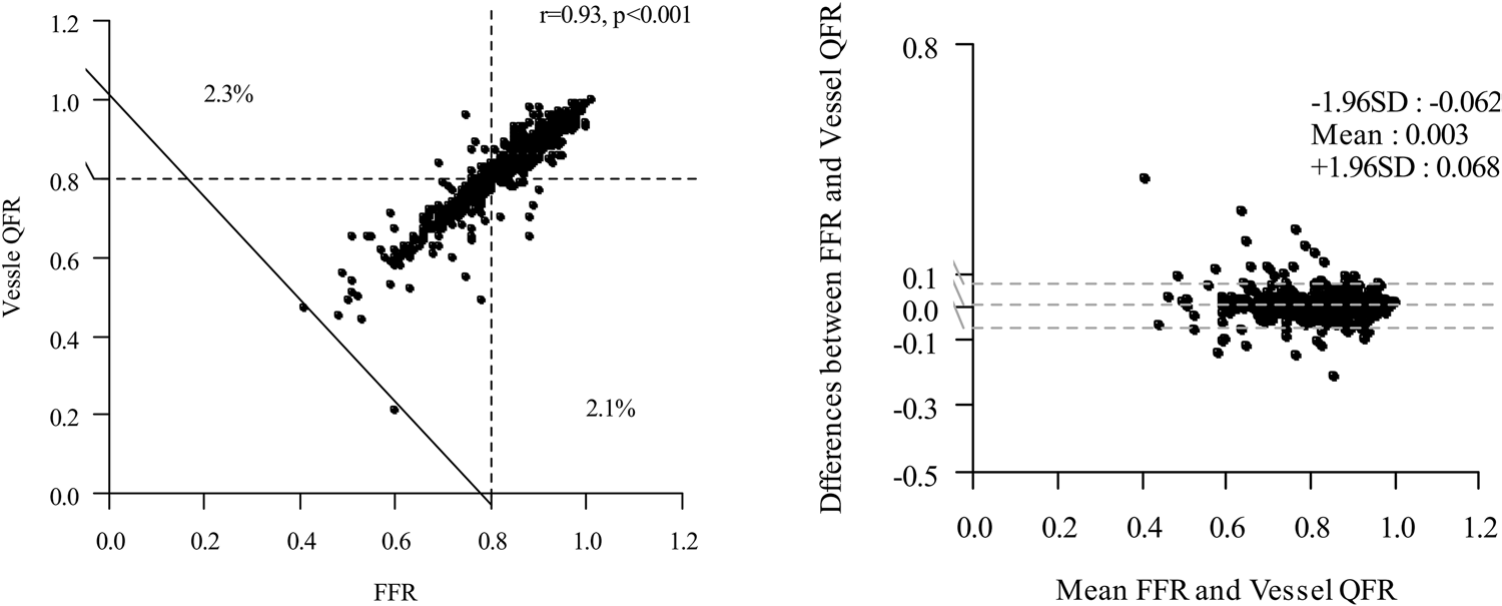
Correlation and agreement between QFR and FFR. Scatter plot shows the correlations between the FFR and QFR in all patients (r = 0.93, Pearson’s rank correlation coefficient). Differences between the FFR and QFR versus the means of the two measurements shown in a Bland–Altman plot.

The anatomical severity of epicardial coronary stenoses was generally intermediate, with a mean percent area stenosis of 64.65 ± 9.72% (Supplemental Fig. 1^1^. The mean FFR was 0.82 ± 0.09, and the FFR was ≤ 0.8 in 408 vessels (37.9%). On the post hoc blind assessment by QFR software, the mean QFR was 0.82 ± 0.09 (Table 1). Figure 2 shows agreement between QFR and FFR. A good correlation (r = 0.93, p < 0.001) and agreement (mean difference: 0.003, limits of agreement: − 0.062 to 0.068) between QFR and FFR were found. The QFR cutoff value that best corresponded to an FFR of 0.80 was a QFR of 0.80 (AUC = 0.98 [95% CI 0.97 to 0.99]). The diagnostic accuracy of the QFR for identifying an FFR ≤ 0.8 was excellent (95.54% [95% CI 94.13 to 96.70%]). The PAS by 3D QCA showed a lower AUC than those of the QFR values (0.69 [95% CI 0.66 to 0.72] vs. 0.98 [95% CI 0.97 to 0.99]). The sensitivity, specificity, PPV and NPV were also higher for QFR compare to PAS (94.12% vs. 45.59%, 96.41% vs. 81.41%, 94.12% vs. 60.0%, and 96.41% vs. 70.98%, P < 0.001 each). The median time to complete QFR was 7.27 min (interquartile range, 5.0 to 9.0).

### Comparison of the diagnostic performance of the QFR in the angina, AMI, borderline FFR and complex lesions subgroups

**Figure 3:**
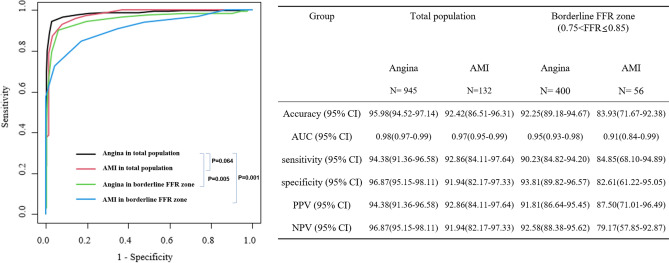
Comparison of the diagnostic performance of QFR for identifying FFR 0.8 in the total population and a subgroup of borderline FFR zone. P values are from the chi-square test or Fisher's exact test for comparing diagnostic accuracy. *AUC* the area under the curve, *CI* confidence interval; other abbreviations in Table 1.

**Figure 4:**
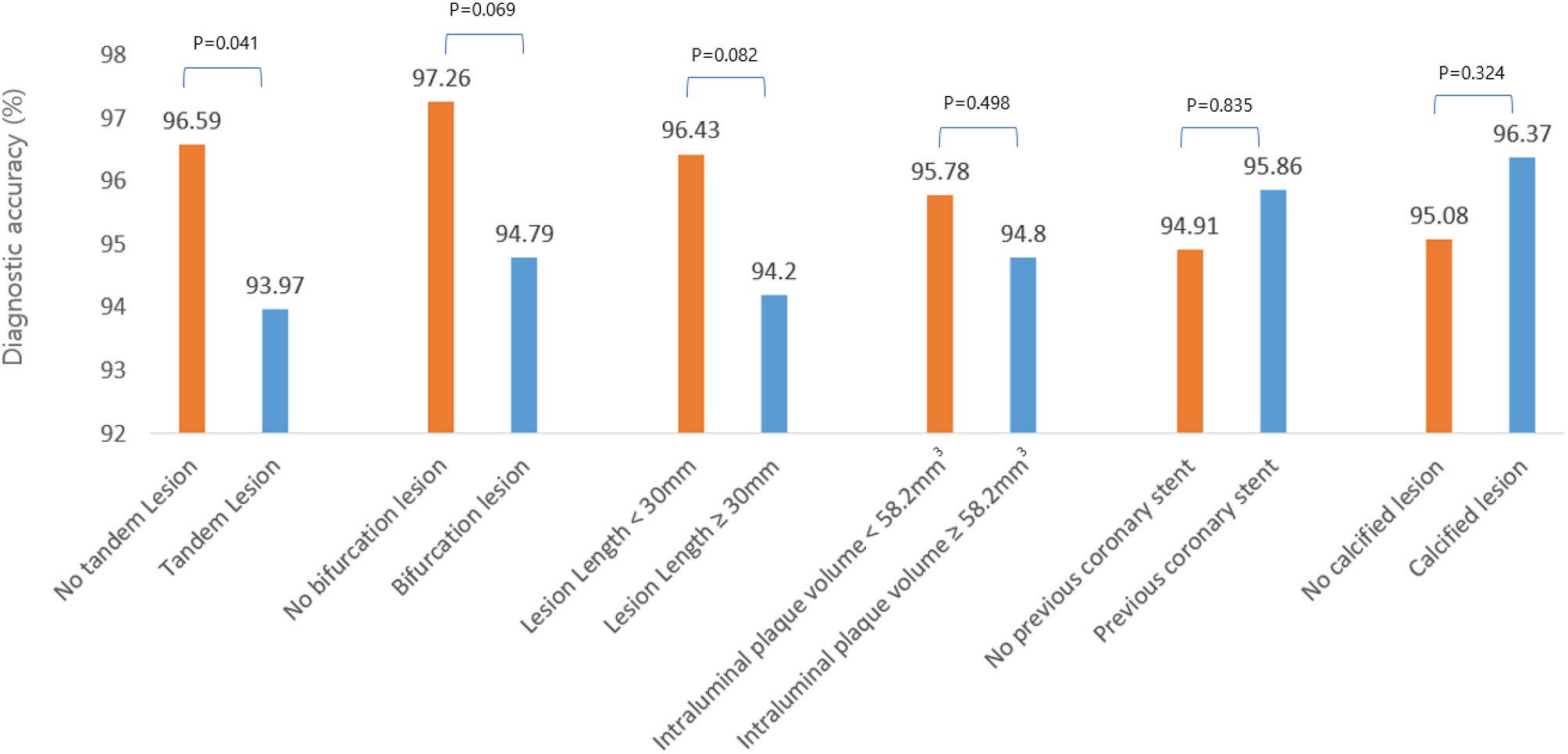
Diagnostic performance of QFR against FFR in different subgroups of complex lesions. The diagnostic accuracy of QFR measured in vessels with tandem lesions was significantly lower than in vessels with single lesions. P values were calculated using the chi-square test or Fisher's exact test.

**Figure 5:**
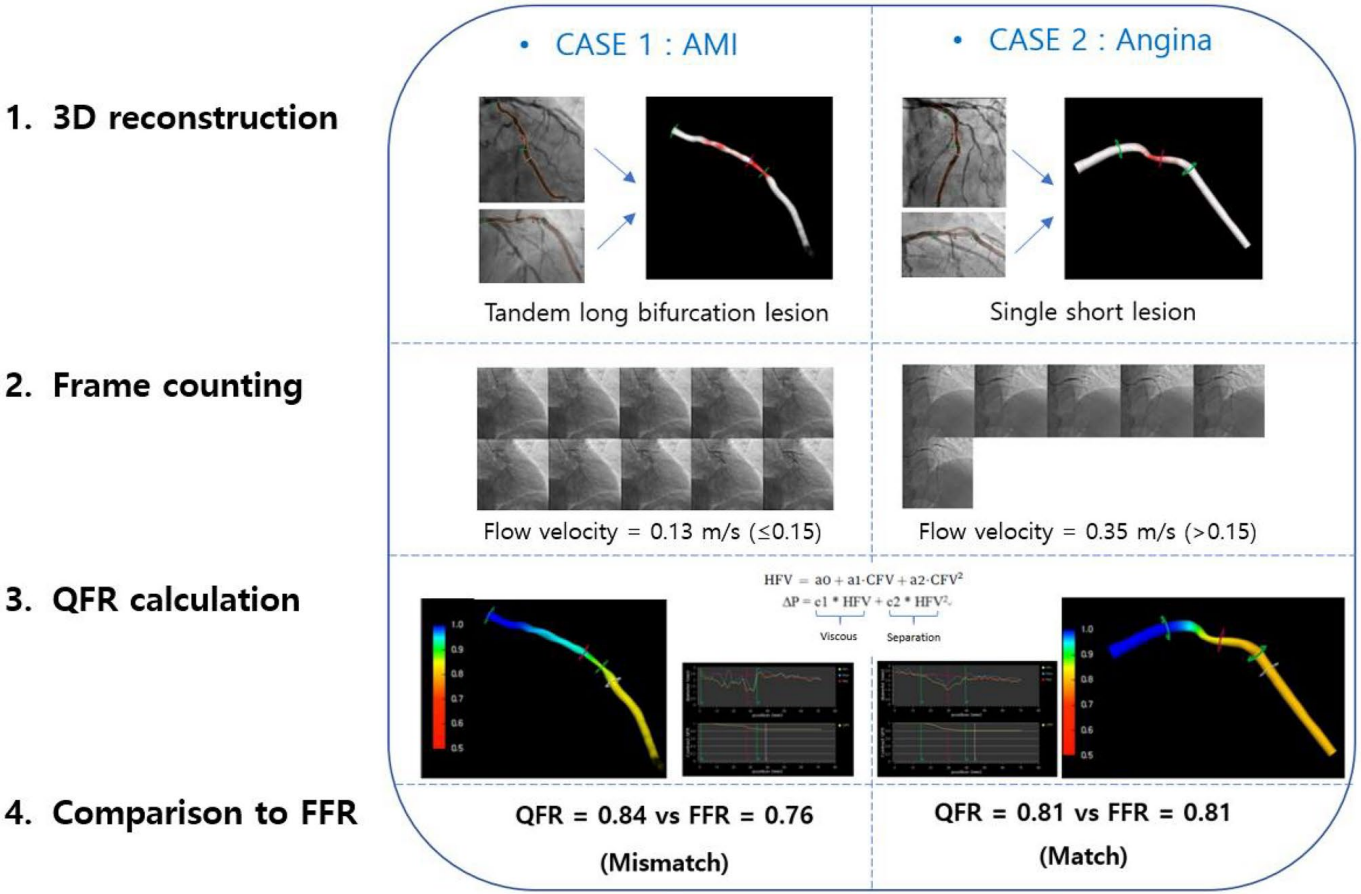
Computation of QFR by coronary angiography in 2 representative QFR-FFR mismatched and matched cases. Case 1: The calculated QFR value in a noninfarct-related artery accompanying lesions with multiple complexities was mismatched with the FFR. Case 2: The calculated QFR for a vessel in a patient with angina accompanying a simple lesion was exactly matched with the FFR. The QFR analysis images were extracted with QAngio XA 3D/QFR, V 1.2 by Medis Medical Imaging, Leiden, The Netherlands.

The diagnostic accuracy, sensitivity, specificity, positive predictive value, and negative predictive value of the QFR for identifying an FFR ≤ 0.8 in the angina population were 95.98%, 94.38%, 96.87%, 94.38%, and 96.87%, respectively. The AUC of the QFR was higher than that of the PAS (0.98 [95% CI 0.97 to 0.99] vs. 0.70 [95% CI: 0.66 to 0.73]). This trend was consistent in the AMI subgroup. (Supplemental Table 1). There was no significant difference in diagnostic accuracy between the angina and AMI groups (Fig. 3, p = 0.064). The diagnostic accuracy of a QFR of ≤ 0.8 for identifying an FFR of ≤ 0.8 in the AMI group was 92.42%, with a sensitivity of 92.86%, a specificity of 91.94%, a PPV of 92.86%, and an NPV of 91.94%. With lesions of the borderline FFR zone (0.75 < FFR ≤ 0.85), the overall diagnostic accuracy of QFR was significantly lower than that of the total population (91.23% [95% CI 88.25 to 93.66%] vs. 95.54% [95% CI 94.13 to 96.70%]; difference: 4.32; p = 0.001). Based on the angina group's diagnostic accuracy in the total population as a reference, the classification agreements for the angina and AMI groups in the boundary FFR area were significantly lower (92.25% [95% CI 89.18–94.67] and 83.93% [95% CI 71.67–92.38]; difference: 3.73 and 12.05; p = 0.005 and p = 0.001, respectively). The diagnostic accuracy of the QFR in the subgroup with tandem lesions was 93.97% (95% CI 91.29 to 96.02%), which was lower than that of the nontandem lesion group (96.59% [95% CI 94.89 to 97.85]; p = 0.041) (Fig. 4). These associations were not significant after Bonferroni-corrected significance threshold (α = 8 × 10 − 3, correcting for 6 tests). The accuracies of QFR in groups with bifurcation lesions and a long lesion length were low, but there was no significance (p = 0.082 and 0.069, respectively). The existence of a large intraluminal plaque volume, a history of previous coronary intervention and calcification did not affect the diagnostic performance of QFR. The process and the results of QFR analysis cases are depicted in Fig. 5, with 2 representative cases. A calculated QFR value in a patient with AMI with tandem long bifurcation lesion was mismatched with the FFR (Case 1). The calculated QFR in a patient with angina with a simple lesion exactly matched the FFR (Case 2).

## Discussion

In this study, we observed the diagnostic performance of QFR in all-comer patients who underwent FFR not only in the angina population but also in the AMI population with noninfarct-related arteries. We conducted QFR assessments for the largest population to date. We only excluded vessels with calibration failure, and our exclusion rate was only 11.8%, which was numerically lower than those in previous reports^13,15^.

The diagnostic accuracy of the QFR with the FFR as a reference in our work was 95.54%, which is the highest accuracy reported among existing studies, possibly because our 2 investigators assessed the QFR after training with more than 300 patients each. After QFR analysis training, we conducted a pilot study (110 cases) before entering the QFR analysis of this registry data. The interobserver variability of QFR were assessed using the intraclass correlation coefficient (ICC). The ICC between the two observers was 0.90 (95% CI: 0.82 to 0.94) indicated excellent agreement. Another possible reason for the good results in our study is that 34.8% of the evaluated vessels had PAS < 50% or > 70% which may have resulted in increased diagnostic power (Supplemental Fig. 1). However, this observation cannot explain all the results because the incidence of vessels (37.8%) with an FFR ≤ 0.8 was comparable to that in previous studies (33% and 34%)^13,15^.

Importantly, this study includes comparison data of the diagnostic performance of the QFR for identifying an FFR of ≤ 0.8 in the AMI and angina groups (Fig. 3). Until recently, the diagnostic performance of QFR was mostly assessed in patients with stable coronary disease. Concern regarding the variability of QFR values under AMI conditions has emerged because the calculation is based on the assumed flow velocity with frame counting, which can be affected by microvascular resistance. The representative cases reflecting this hypothesis are briefly and prominently presented in Fig. 5. Case 1 describes that the QFR value can be mismatched with the FFR in vessels that are noninfarct-related arteries in AMI conditions and contain multiple lesion complexities, such as tandem long bifurcation lesions. Case 2 describes that QFR matches well with FFR values and has very high diagnostic power in patients with only single short lesions. QFR analysis applies correction values using flow velocity information by the frame count method as well as geometry information of vessels. A low flow velocity may indicate high microvascular resistance and damage, especially in AMI conditions.

From our cohort, we observed that the accuracy of the QFR in the AMI group was relatively lower than that in the angina group without a significant difference (92.42% [95% CI 86.51 to 96.31%] vs. 95.98% [95% CI 94.52 to 97.14%]; difference 3.55; p = 0.064). However, in the conventional ‘borderline FFR zone’ group (FFR 0.75–0.80) accompanying AMI, the diagnostic accuracy of the QFR for FFR was significantly lower than that in the total population with angina. (83.93% [95% CI 71.67 to 92.38%]; difference 12.05 [95% CI 2.35 to 21.75]; p = 0.001) (Fig. 3). The condition accompanying both AMI and the borderline FFR zone showed the lowest QFR diagnostic accuracy in our data. These outcomes are consistent with the results of previous studies^18,19^. Under AMI conditions, transient microvascular dysfunction may occur due to microembolization, inflammation, myocardial edema, or necrosis, especially when the no-reflow phenomenon occurs^20^. This microvascular damage is not limited to the infarct-related artery but may also extend to the noninfarct related artery territory^21^. In previous studies, lower intracoronary measured coronary flow velocity reserve and higher minimal microvascular resistance were observed in the infarcted and noninfarcted regions during AMI than at follow-up^22–24^. It may cause submaximal hyperemia during the FFR test, which may interfere with the measurement of stenosis severity indices. Besides, this may result in the low diagnostic accuracy of QFR to FFR for AMI patients.

**Figure 6:**
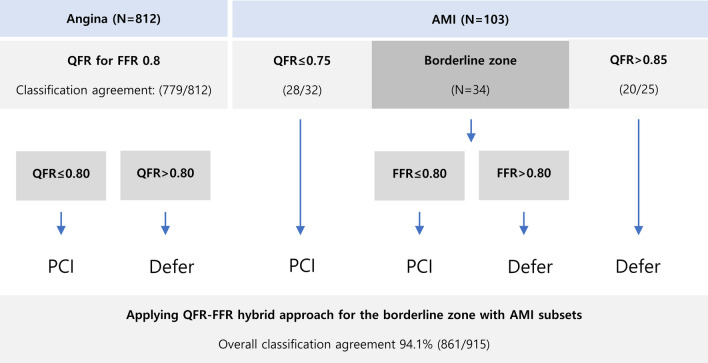
QFR-FFR Hybrid approach strategy.

We suggest a QFR-FFR hybrid approach for these subsets with low diagnostic power (Fig. 6). Applying to our data, with FFR measurement for AMI patients with borderline QFR zones, the overall classification agreement of the proposed QFR-FFR hybrid approach is 94.1%. QFR uses the 3D rendering image once processed from angiography images and then performs computational calculations again. Indirect diagnostic equipment with multistage calibration should be used in a manner that minimizes the risk of error.

Another strength of this study was that we assessed the anatomical complexities of stenosis lesions to identify specific subsets with lower diagnostic accuracy. The diagnostic accuracy of QFR against FFR in vessels with tandem lesions was lower than that in vessels with nontandem lesions (93.97% vs. 96.59%, p = 0.041, not correcting for multiple testing) (Fig. 4). There was a trend toward lower diagnostic performance in bifurcation lesions and long lesions (94.79% vs. 97.26% and 94.20% vs. 96.43%; p = 0.069 and p = 0.082, respectively). The difficulties imposed by hyperemia and complex geometry in tandem lesions may have hindered the diagnostic performance of QFR analysis using CFD calculations. The degree of pressure drop across stenosis depends on the (1) stenosis severity and (2) the amount of flow across it^25,26^. First, when calculating the effect of stenosis severity on pressure loss in CFD, both fiction loss at a proximal part and loss due to spatial variations at the distal portion of stenosis should be considered (Fig. 5). At this time, the stenosis pattern's geometrical diversity can affect the low accuracy of the calculation^27^. It requires much calibration and is less accurate when reconstructing the stenosis geometry twice in vessels with tandem lesions. Second, QFR using the resting contrast flow velocity obtained from frame count has difficulty accurately inferring hyperemic flow velocity between each stenosis. Further investigations with large volume studies will be needed on this subject.

In this study, we paid attention to setting exclusion criteria. Our centers have been adopted a fixed angle setting of a CAG projector and prohibit table movement. Therefore, it showed a lower exclusion rate (11.8%) than other studies (12 to 15%)^13,14^. It is essential to standardize the basic CAG protocol first when applying QFR for universal use in clinical practice. If we set the CAG protocol to fix the designated angles of view with a difference of more than 25 degrees, without panning, and without magnification during recording, we could lower the exclusion criteria to less than 10%.

### Limitations

This study has several limitations. First, a large proportion of the vessels were assessed to have mild coronary stenotic lesions (PAS < 50%) or severe stenosis lesions (PAS > 70%), even though the clinicians decided to measure the FFR, believing that the patient had angiographically intermediate stenosis. It may have increased the overall diagnostic accuracy of QFR 0.8 against FFR 0.8. However, we assessed QFR as a post hoc analysis in all-comer patients who underwent FFR evaluations in the real world, which is more meaningful in some ways. At present, FFR has been performed not only for conventional intermediate (50–70%) stenosis but also for a broad spectrum of 40 to 90% stenotic lesions in the real world. Considering the presence of low diagnostic power of the borderline FFR zone in a specific subset, we think it may be more helpful to use QFR selectively to filter our patients whose QFR values do not correspond to a borderline FFR zone. Second, the analysis was performed with only 2 physicians, which could not ensure that there was no interobserver variation. A standardization of the assessment method with automatic analysis using artificial intelligence will be needed in the future. In addition, further evidence with a large study is still needed to investigate the role of QFR in specific subgroups of patients with microcirculatory disease, arrhythmias, culprit lesions with AMI and other complicated lesions.

## Conclusion

In this study, we observed that QFR showed excellent diagnostic performance in detecting functional ischemia, which was comparable to that of FFR in a real-world all-comer population cohort. The deferral based on negative QFR values could be as effective as the decision made by the FFR values in populations not only with stable angina but also with AMI. However, we observed a lower diagnostic performance of QFR at the borderline FFR zone in patients with AMI, which requires the hybrid approach.

## Supplementary Information

Supplementary Information.
