# Supplementary material for: Influence of lesion and disease subsets on the diagnostic performance of the quantitative flow ratio in real-world patients
Source: Sci Rep. 2021 Feb 4;11:2995. doi: 10.1038/s41598-021-82235-y (PMC7862355; doi:10.1038/s41598-021-82235-y)

**RESEARCH**

**Influence of lesion and disease subsets on the diagnostic performance of the quantitative flow ratio in real-world patients**

Kwan Yong Lee^1^, Byung-Hee Hwang^2^, Moo Jun Kim^3^, Eun-Ho Choo^2^, Ik Jun Choi^1^, Chan Jun Kim^4^, Sang-Wook Lee^5^, Joo Myung Lee^6^, Mi-Jeong Kim^1^, Doo Soo Jeon^1^, Wook Sung Chung^2^, Ho-Joong Youn^2^, Ki Jun Kim^7^, Myeong-Ho Yoon^8^, Kiyuk Chang^2^

*1. Department of Cardiology, Incheon St. Mary’s Hospital, The Catholic University of Korea, Incheon, Republic of Korea; 2. Department of Cardiology, Seoul St. Mary’s Hospital, The Catholic University of Korea, Seoul, Republic of Korea; 3. Cardiovascular Research Institute, Seoul St. Mary’s Hospital, The Catholic University of Korea, Seoul, Republic of Korea; 4. Department of Cardiology, Uijeongbu St. Mary’s Hospital, The Catholic University of Korea, Uijeongbu, Republic of Korea; 5. School of Mechanical Engineering, University of Ulsan, Ulsan, Republic of Korea; 6. Department of Cardiology, Department of Internal Medicine, Heart Vascular Stroke Institute, Samsung Medical Center, Sungkyunkwan University School of Medicine, Seoul, Republic of Korea; 7. Department of Radiology, Incheon St Mary’s Hospital, The Catholic University of Korea, Incheon, Republic of Korea; 8. Department of Cardiology, Ajou University Medical Center, Suwon, Republic of Korea*

****Corresponding author: Byung-Hee Hwang, MD***

*Division of Cardiovascular Center and Cardiology, Seoul St. Mary’s Hospital, College of Medicine, The Catholic University of Korea, 222, Banpo-daero, Seocho-gu, Seoul, 06591, Republic of Korea*

*Fax: +82 2 2258 1142; Tel.: +82 2 2258 1139, Email: hbhmac@catholic.ac.kr*

**Supplemental materials**

Supplemental Table 1. Comparison of the diagnostic performance of the QFR and PAS values in the total population, patients with angina, and patients with acute myocardial infarction.

|  |  |
| --- | --- |
|  | AUC (95% CI) |
|  | for FFR≤0.8 |
| *Total pts (n=1077)* |  |
| QFR | 0.98 (0.97-0.99) |
| PAS | 0.69 (0.65-0.72) |
| *Angina (n=945)* |  |
| QFR | 0.98 (0.97-0.99) |
| PAS | 0.69 (0.64-0.73) |
| *Acute myocardial infarction (n=132)* |  |
| QFR | 0.97 (0.95-1.00) |
| PAS | 0.62 (0.53-0.72) |
| AUC indicates the area under the curve; QFR, quantitative flow ratio; PAS, percent area stenosis. | |
|  |  |
|  |  |
|  | |

Supplemental Figure 1. Distributions of the PAS, FFR, and vessel QFR in all lesions.


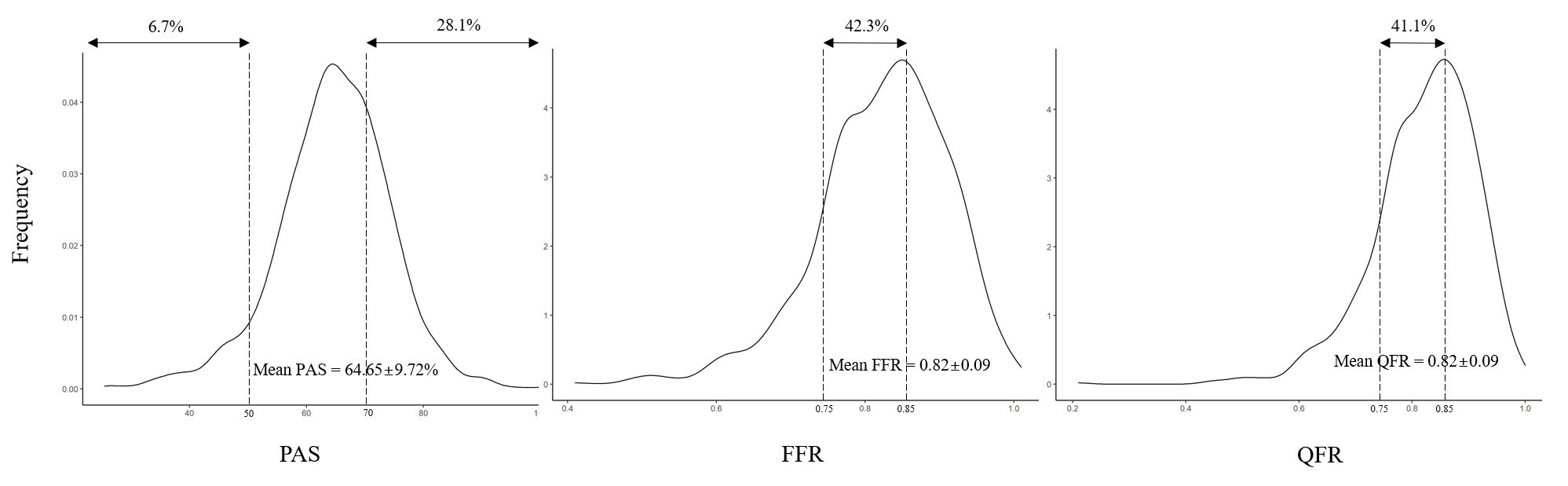

Supplement: Supplementary file 1 — Supplementary Information. [file 41598_2021_82235_MOESM1_ESM.docx]
